# Alkalinity and Its Consequences for the Performance of Steel-Reinforced Geopolymer Materials

**DOI:** 10.3390/molecules25102359

**Published:** 2020-05-19

**Authors:** Andreas Koenig, Hitham Mahmoud, Oliver Baehre, Frank Dehn

**Affiliations:** 1 Dental Prosthetics and Materials Science, Leipzig University, 04275 Leipzig, Germany; 2 Else Kooi Lab., Faculty of Electrical Engineering, Mathematics and Computer Science, Delft University of Technology, 2628 CT Delft, The Netherlands; h.m.aminhassan@tudelft.nl; 3 Chemistry Department, Faculty of Science, Zagazig University, Zagazig 44519, Egypt; 4 Institute of Mineralogy, Crystallography and Materials Science, Leipzig University, 04275 Leipzig, Germany; oliver.baehre@uni-leipzig.de; 5 Institute of Concrete Structures and Building Materials, Karlsruhe Institute of Technology, 76131 Karlsruhe, Germany; frank.dehn@kit.edu

**Keywords:** geopolymer concrete, alkalinity, micro X-ray computer tomography (µXCT), steel reinforcement corrosion

## Abstract

This paper investigates the development of the alkalinity and its impact on carbon steel reinforcement embedded in alkali-activated fly ashes (AAFA) and alkali-activated fly ashes with ten percentage mass (wt%) of blast furnace slag (AAFAS)-based materials (geopolymer–GP). The pH analysis of eluates indicates a remarkable decrease of alkalinity in AAFA and AAFAS in the first hours of the geopolymerization process. Phenolphthalein solution and pore solution tests on concretes also show a sharp decrease of alkalinity with increased *Ca* content in the binder due to carbonation. Micro X-ray computer tomography (µXCT) and electrochemical techniques indicate that the changed pH in the GP systems was accompanied by a decrease in the corrosion rates of steel reinforcement when compared to ordinary Portland cement (OPC) systems. In contrast to calcite and vaterite, which were detected in OPC and AAFAS after a carbonation process, only sodium carbonate natron was determined at lower levels in AAFA by X-ray diffraction (XRD).

## 1. Introduction

Alkali-activated binders (AABs)/geopolymer binders (GPs) are increasingly in the focus of research and development (one in ten articles in the best-known cement and concrete journals refers to AABs or GPs [1]). AABs/GPs show advantageous properties with regard to environmental protection (air pollutant reduction, resource and energy conservation, reuse of waste materials [2,3,4,5]), improved chemical resistance [6,7,8,9,10], and adequate mechanical performance [2,11,12].

In most current literature, GPs are defined as low *Ca* AABs with high *Al* and *Si* content (e.g., fly ash, metakaolin). The notion AAB is defined as a generic term [1,13]. Dependent on the *Ca* content, different groups of reaction products, like N-A-S-H (low *Ca* content), C-A-S-H, C-(N)-A-S-H, and C-S-H(I) (very high *Ca* and low *Al* content) phases will be formed (see also STAR [2]). The threshold of the *Ca* content can be defined based on the chemical composition of the raw material, for example fly ash (7 wt% according ASTM C618, type F or 10 wt% according to EN 450-1), the investigated crystallographic structure of the reaction products (<20 wt% [14]) or the performance of the concretes/mortars (10 wt% [1,13]).

Alkalinity is one of the most important material parameters in concrete technology. This value specifies the type of reinforcement materials required [15,16] and influences the long-term stability of the solid phases. In the future, the relevance of the leaching behavior of building materials [17,18] in general, and for AAB in particular [19,20], will increase with regard to their environmental compatibility.

In concretes based on ordinary Portland cements (according EN 197-1), alkalinity in the microstructures results from the hydration product portlandite (*Ca(OH)_2_*) as well as the release of sodium and potassium ions from the clinker [21]. In the absence of carbonation and/or leaching, alkalinity in fresh Portland cement concrete is related to the saturation of the concrete pore solution with *Ca(OH)_2_*, which causes the precipitation of *Ca(OH)_2_* in crystalline form (portlandite) within the pore, enhancing the pH of concrete to approx. >13 [21,22]. The alkalinity buffer in the microstructure depends indirectly mainly on the clinker content in ordinary Portland cements and directly on the saturated *Ca(OH)_2_* within the pore solution. From a thermodynamic point of view, ordinary Portland cement reaction products (such as ettringite, portlandite, C-S-H) are stable only in the long term if the pH value is higher than ten [21,22,23,24,25,26,27,28].

The alkaline environment promotes the generation of an electrochemically stable protective passive layer on the embedded steel surfaces (passivation) when the pH of the concrete is higher than ten [29]. The permeability of the bulk concrete matrix generally increases under leaching conditions. This increase in permeability is very slow in ordinary Portland cement (OPC) concretes because of the low water solubility of the buffering *Ca(OH)_2_* (CH). At the same time, *CO_2_* from the atmosphere penetrates in the pore matrix of the concrete and C-S-H, CH, monosulfphate (AFm), and tricalciumsulphate (AFt) react to different kinds of *CaCO_3_* phases (aragonite, calcite, vaterite) and *SiO_2_* gels. The chemical reaction of *CO_2_* with CH reduces the alkalinity, and the new reaction products change the permeability (by carbonation-induced shrinkage, changing total and effective capillary porosity). Additionally, the hydration of cement with a high content of blast furnace slag (>50 wt%) and/or pozzolans results in the formation of C-S-H with a very low C/S ratio that is resistant to carbonic acid [30,31,32].

In the case of AAB, some authors [33,34,35,36] reported an improvement in the resistance of the reinforcement to corrosion thanks to the prevention of the ingress of harmful aggressive agents (e.g., chlorides, *CO_2_*), which could initiate steel corrosion. On the other hand, the data on alkali-activated blast furnace slag (AAS) binders indicate a greater susceptibility to carbonation in accelerated lab tests (1% to 3% *CO_2_*) compared to OPC binders [37]. This depends mainly on the binder content [38] and the method of activation [39]. In the literature, the higher carbonation rate of AAB is attributed mainly to the low activation concentration and the absence of CH, which leads to a very low buffer capacity [40] and vulnerable C-S-H [41].

Nevertheless, long-term experiences under normal CO_2_ concentrations with AAS concrete pipes (based on blast furnace slag) showed a pH value of 11.5 [41] after 34 years of service life and with slag-fly ash AAB concrete samples after eight years a pH of approximately 11.4 in noncarbonated areas [42]. In GP-based concrete systems, only alkali ions in the pore solution can react with *CO_2_*, forming different types of carbonate hydrate, depending on temperature, humidity, and *CO_2_* concentration [43,44,45,46]. Bernal et al. [44] and Cyr and Pouhet [22] have reported that alkali-activated fly ash and metakaolin-based concrete systems showed very high carbonation rates under higher *CO_2_* concentrations (≥7 vol.-%) compared to OPC-based concrete. The authors explain the mode of action by means of different kinds of carbonate phases occurring in accelerated lab tests with *CO_2_* concentrations >1 vol.-%, which are not representative for carbonation products under normal conditions. Aggravating to carbonation, Neves et al. [46] have concluded that, in the case of geopolymer concrete, the drop in the pH of the geopolymer concrete pore solution was mainly related to the geopolymer process and was not only due to carbonation. In addition, Lloyd et al. realized that the alkali leaching process induced a drop in the pH value in the proximity of the embedded steels [47].

Most investigations of the steel reinforcement durability in GP and AAB concretes are focused on corrosion processes caused by the ingress of chlorides [48,49,50,51,52,53,54,55], and only few investigate carbonation [56,57] and almost none look at leaching or geopolymerization processes.

The currently available results are inconsistent with one another. In the case of chloride-induced corrosion, for example, the investigations by Monticelli et al. [54], Tittarelli et al. [50], and Farhana et al. [56] have shown greater protection for steel reinforcement bars in low-calcium fly ash-based GP-mortars/concrete compared to cementitious materials, whereas Babaee and Castel [48] have demonstrated comparable protection, and Gunasekara et al. [55] lower protection.

There is still a lack in knowledge related to the electrochemical and corrosion response of steel reinforcement embedded in AAB and GP. Consequently, there is still an open debate in relation to the durability of reinforced AAB-based concretes.

The aim of the present study was to provide further evidence and improved understanding of AAB carbonation and leaching and of their effect on the electrochemical response and corrosion resistance of carbon steel reinforcement. The study was carried out by combining geopolymer durability investigations and microstructure changes with reinforcement electrochemical measurements.

## 2. Results

### 2.1. Carbonation Resistance of Concretes under Normal Conditions

**Figure 1:**
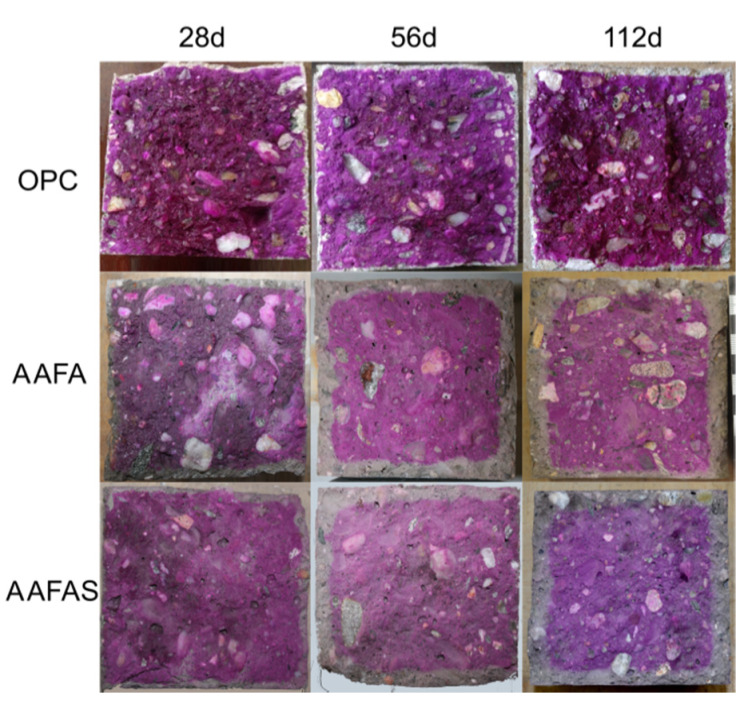
Fractured surface of concrete cubes (100 mm^3^) after 28, 56, and 112 days in 20 °C, 65% rH, 0.04 vol.-% *CO_2_*.

**Figure 2:**
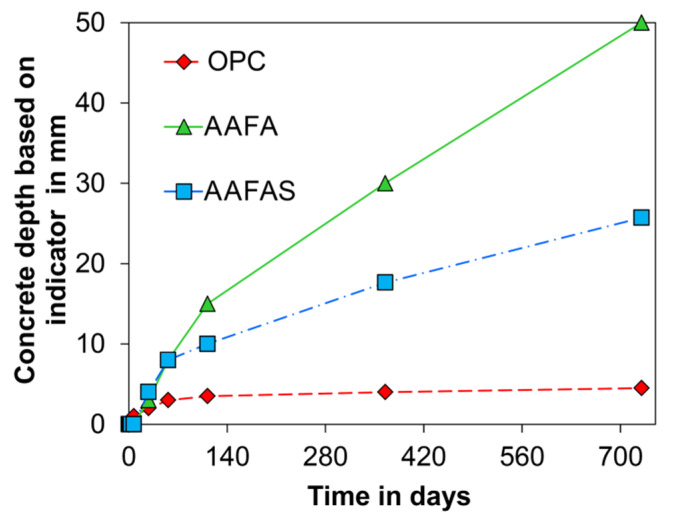
Depth of non-pink colored material in concrete were stored at 20 °C/65% rH, 0.04 vol.-% *CO_2_*.

After seven days under standard climate conditions (20 °C/65% rH/0.04 vol.-% *CO_2_*), a discolored layer was observed in the AAB and OPC concrete samples. With increased storage time, the OPC concretes showed a discolored layer and the AAB concretes a pink colored layer near the surface of the cross section when a phenolphthalein solution was used (Figure 1). The zoning in the cross sections increased linearly in the FA, in the FAS as a slightly square root function, and in the OPC concretes as a significant square root function (described by a power law) until the age of 730 days (Figure 2). The FA sample showed no dark pink colored layer but did present single light pink colored areas in the cross sections after 730 days.

**Figure 3:**
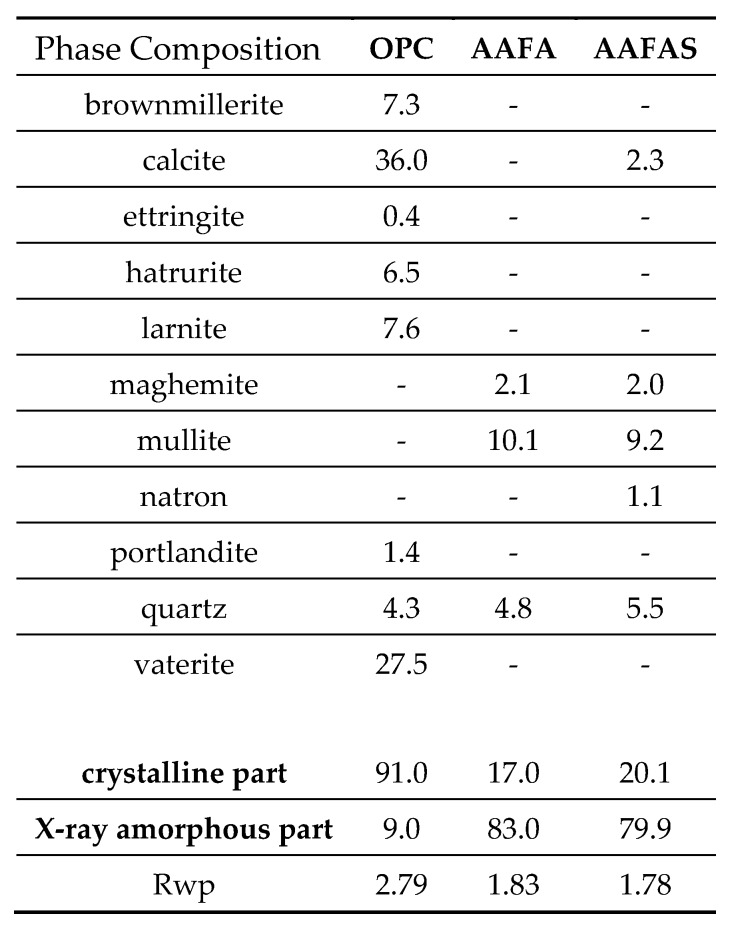

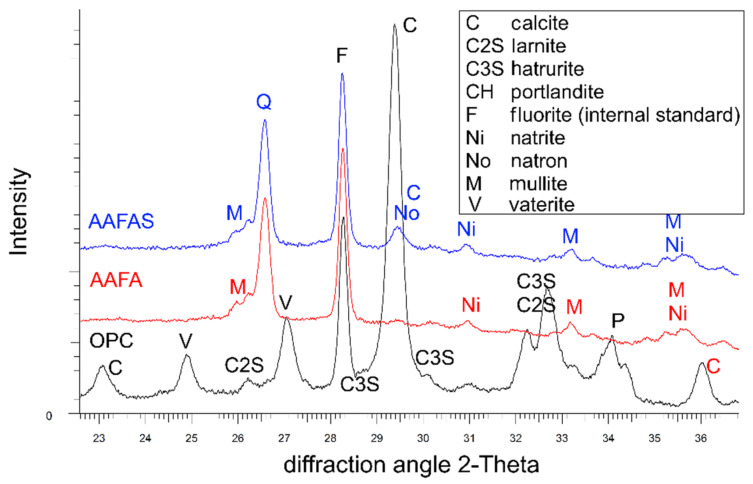
Phase composition (**up**) and diffraction patterns (**down**) of hardened binder pastes stored as powder (<125 µm) for 112 days (20 °C/ 65% rH/0.04% *CO_2_*).

The type of carbonation products depends on the reaction products based on the binder system and the *CO_2_*-concentration. Very high calcite (*CaCO_3_*) content and less content of vaterite (*CaCO_3_*) contents were detected radiographically in OPC samples. In both GP samples (AAFA, AAFAS), natron (*Na_2_CO_3_·10H_2_O*) was detected in a low concentration. A significant reflex was obtained at 29.4 2-Theta only in the AAFAS sample; this can be assigned to calcite. The diffraction patterns, which were used for Rietveld refinement, are shown in Figure 3.

**Figure 4:**
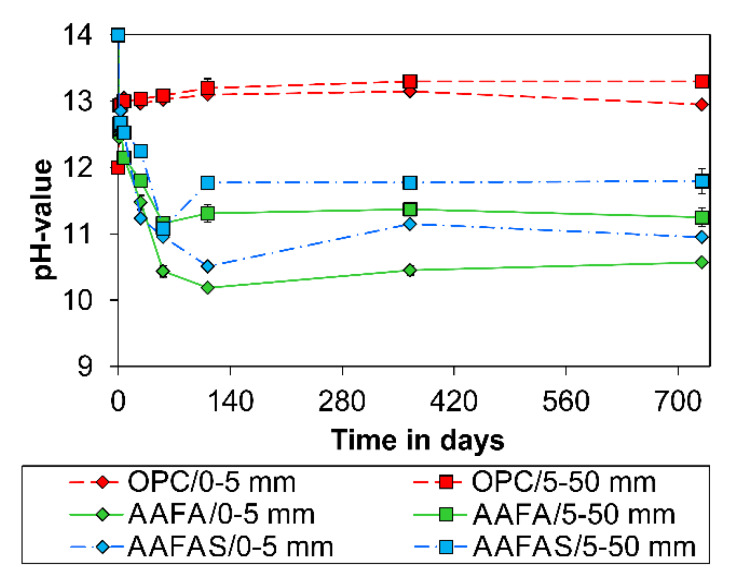
Development of pH value at different depths in concrete under 20 °C/65% rH/0.04 vol.-% CO_2_.

**Figure 5:**
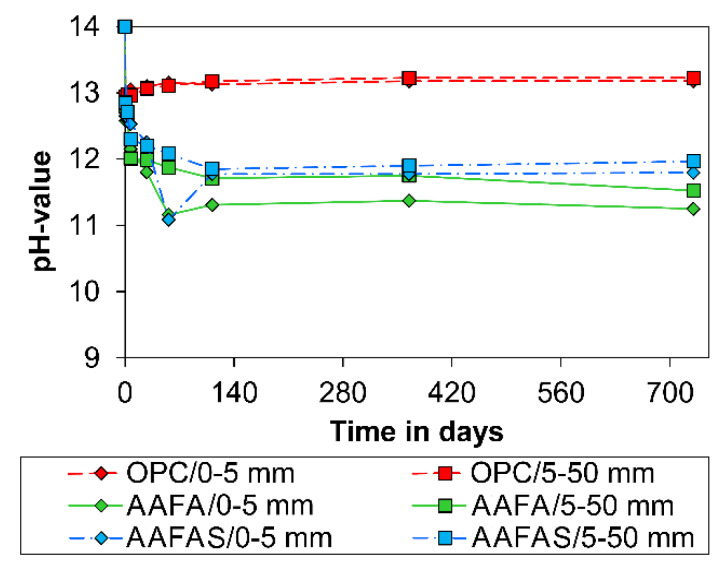
Development of pH value at different depths in concrete under 20 °C/65% rH/0.00 vol.-% *CO_2_*.

On the other hand, the pH value of the eluates produced from concrete samples depends on the distance from the sample surface (Figure 4) and the *CO_2_* concentration (Figure 5).

### 2.2. Eluate Monitoring Tests to Examine the Influence of the Hydration and Polymerisation Reaction on the Alkalinity

To determine the development of alkalinity in AAB at different time intervals (1 and 24 h), the eluate pH values were measured. With rising *NaOH* concentration in demineralized water, the pH value will increase. However, in the presence of blast furnace slag (S), fly ash (FA), and fly ash with blast furnace slag (FAS), different responses are monitored, which are related to the consumption of the *OH^−^* in the binder activation process.

**Figure 6:**
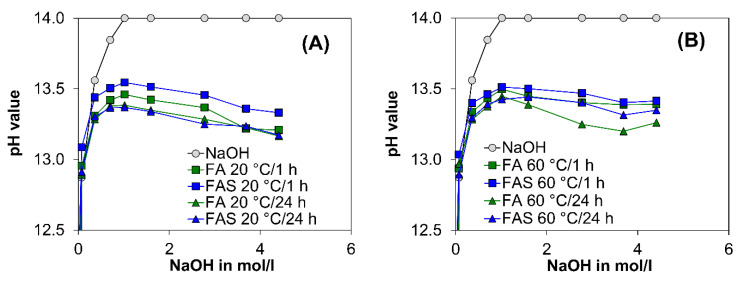
Eluate results of pure NaOH without raw materials (marked as circles), with fly ash (FA) and with 90% fly ash and 10% slag (FAS), after 1 h (marked with square) and after 24 h (marked with triangle), at 20 °C (**A**) and at 60 °C (**B**).

Figure 6 shows the variation in the eluate pH of the solutions containing *NaOH*, FA, and FAS after 1 and 24 h. For a comparative study, two different temperatures (20 and 60 °C) were considered. The data in Figure 6 indicates that in binder-free solutions (black circle), with the addition of *NaOH* (1 mol/L), the eluate pH value varied from a value of approximately 13 up to nearly 14. Nevertheless, at the same temperature and in the presence of FA and FAS, the eluate pH reached lower values than in the binder-free *NaOH* solutions (see Figure 6).

At a temperature of 20 °C, when the *OH^−^*-concentration increased to 2 mol/L, the eluate pH values decreased as *OH^−^*-concentration increased. This response was also observed in solutions at a temperature of 60 °C. The pH values of the eluates with FA reached a lower pH value than solutions with FAS, after 1 h at 20 °C and 60 °C. After 24 h, FA and FAS eluate pH values at 20 °C were similar. However, at 60 °C, FA eluate solutions showed the lowest pH values.

Long-time storage (four weeks) showed a constant pH value in the range of 11 to 11.5 for FA and 11.5 to 12.0 for FAS.

### 2.3. Accelerated Carbonation and Leaching Tests on Mortars with Steel Reinforcement to Examine Corrosion Behaviour

#### 2.3.1. Electrochemical Investigations

*Reference—Before accelerated stress*

Two reinforced mortar samples were considered in parallel in order to evaluate the electrochemical response of embedded steels in different AAB and OPC mortars when exposed to accelerated carbonation and leaching.

For comparative purposes, the electrochemical response of the two mortar series was measured before carbonation or leaching conditioning, using non-destructive electrochemical tests (EIS and Rp).

**Figure 7:**
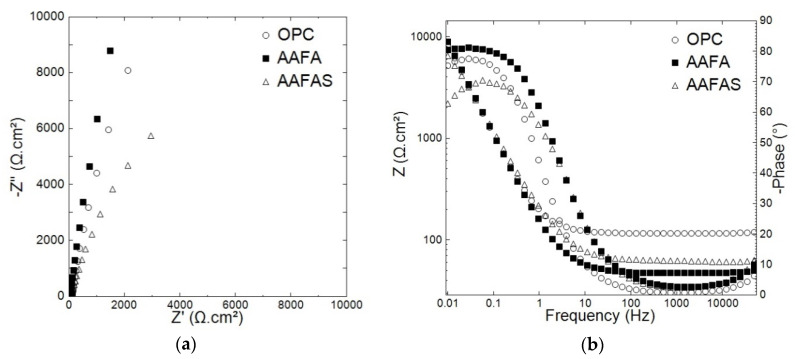
Nyquist (**a**) and Bode (**b**) diagrams of carbon steel embedded in OPC, AAFA, and AAFAS mortars cured for 35 days and before carbonation/leaching.

Figure 7a shows the EIS response of embedded steels in OPC, AAFA, and AAFAS mortars after 35 days of curing (28 days in control conditions and 7 days in lab conditions). The data displayed in Figure 7a shows that at low frequency (lower than 10 Hz), the steel reinforcement embedded in AAFA mortars displays the highest overall-impedance values (Figure 7). Nevertheless, the lowest impedance values were measured in AAFAS samples. Concerning the mortar barrier impedance, at the high frequency limits (higher than 100 Hz), the impedance of OPC mortars barrier was nearly one order of magnitude higher than that of AAB mortars (Figure 7). The lowest mortar barrier impedance was measured in AAFA.

*Accelerated carbonation tests*

**Figure 8:**
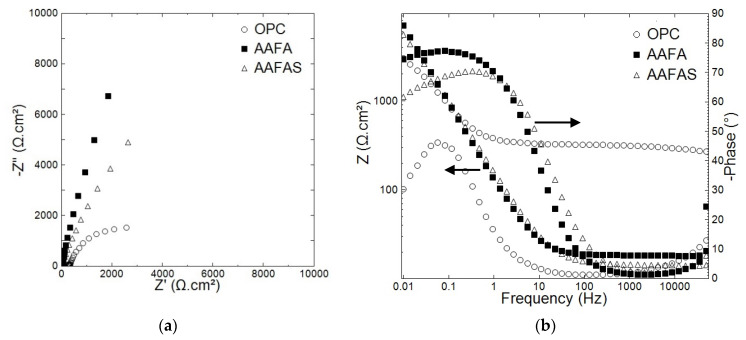
Nyquist (**a**) and Bode (**b**) diagrams of carbon steel embedded in OPC, AAFA, and AAFAS mortars exposed to carbonation for 21 days (1% *CO_2_*, 66% rH).

The EIS scan of the mortar samples after carbonation in a *CO_2_* chamber for 21 days (1% *CO_2_*, 66% rH) is shown in Figure 8. As expected, the carbonation caused the overall impedance values of all tested samples to be lower than for the control cases before carbonation (Figure 7). However, the carbonation-induced corrosion of the embedded reinforcements was only detected in OPC samples. The corrosion response of steel reinforcement was predicted by the resistivity response of the embedded reinforcement in OPC samples, where the phase angle at lower frequency was lower than 40° (Figure 8a). Nevertheless, in the case of the AAFA and AAFAS, more capacitive behavior than OPC samples was monitored (Figure 8a).

The highest impedance values at high frequency limits were measured in AAFA concrete. These results indicate the corrosion resistance of the reinforcement steel. AAFA samples showed higher impedance than AAFAS and OPC.

**Figure 9:**
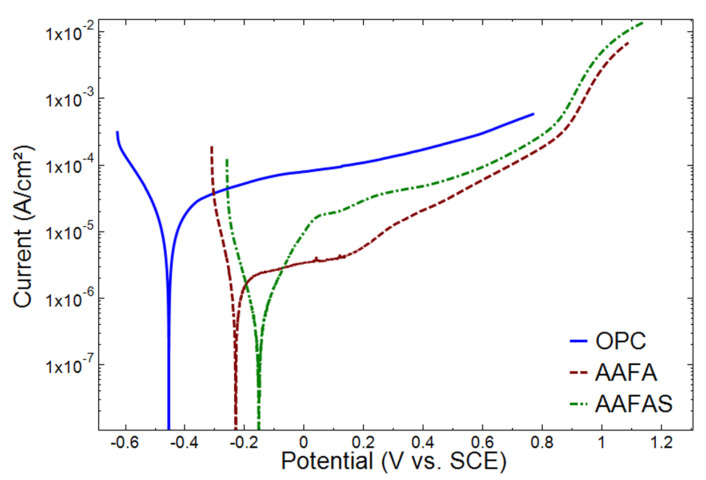
Potentiodynamic polarization (PDP) curves of steel reinforcement embedded in OPC, AAFA, and AAFAS mortars exposed to carbonation for 21 days (1% *CO_2_*, 66% rH).

In order to obtain more detailed information about the corrosion behavior of carbon steel reinforcement embedded in the tested concretes, the Potentiodynamic Polarization (PDP) curves of steel reinforcement in OPC, AAFA, and AAFAS concrete, were considered. The PDP curves of steel reinforcements embedded in OPC, AFFA, and AAFAS concrete are depicted in Figure 9.

The PDP data (Figure 9) indicate the corrosion potential of steel samples embedded in OPC (−0.46 V vs. SCE), AAFA (−0.22 V vs. SCE), and AAFAS (−0.18 V vs. SCE) concretes after accelerated carbonation. The measured cathodic potential of OPC samples was related mainly to the corrosion onset and corrosion propagation induced by the carbonation and pH drop near to the surface of the steel. In the literature, a potential of −0.273 V vs. SCE is given as the potential threshold for corrosion initiation for carbon steel reinforcement in an aggressive environment [58]. Both reinforcement steels in the cases of alkali-activated concrete presented higher corrosion potential than −0.273 V vs. SCE, which in turn indicates the higher corrosion resistance of OPC samples.

Furthermore, the corrosion current density and the passivation current for OPC samples were higher than in AAFA and AAFAS (Figure 9). Although more anodic current was measured in AAFAS samples, the steel reinforcement in AAFAS concrete showed a higher current than in AAFA samples. This confirms the passivation properties of the passive film formed on the steel reinforcement in contact with the pore solution in AAFA concrete. Both AAB mortars had single slight colored areas after the accelerated carbonation but did not show strong layers as in OPC samples near or at the ends of the steel bars.

*Accelerated leaching tests*

Parallel to the carbonation samples, other samples of the same age were exposed to leaching conditions after the last four days of testing conditions in order to investigate the effect of leaching on the electrochemical response of the tested steels in GP concretes and to compare it with reference OPC concretes.

**Figure 10:**
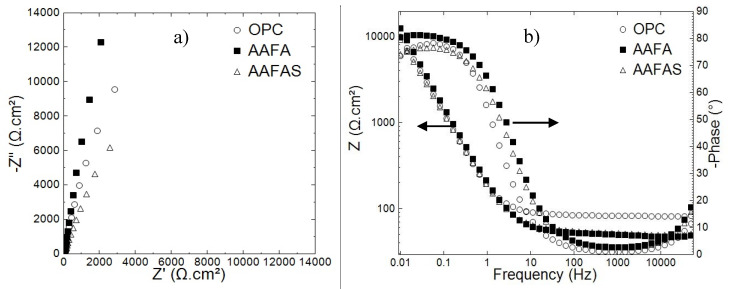
Nyquist (**a**) and Bode (**b**) diagrams of embedded steels in OPC, AAFA, and AAFAS mortars exposed for 4 days at the end exposure conditions.

After 28 days of curing and 24 days under lab conditions, the mortar samples were immersed in deionized water for 4 days. Figure 10 shows the Nyquist and Bode diagrams of the tested samples.

Similar EIS trends to those previously measured under carbonation conditions were observed for the reinforced mortars after leaching; see Figure 7 and Figure 10. The reinforced steels embedded in AAFA showed the highest impedance magnitude at a low frequency limit. The lowest overall impedance values were monitored in embedded steels in AAFAS mortars (Figure 10).

The leaching process affected the mortar barrier impedance values, measured at a high frequency limit. Compared to the control results (shown in Figure 7), relatively low impedance magnitudes were measured after the leaching process. This decrease in the impedance values was relevant in OPC and AAFAS mortars; however, the AAFA barrier impedances values do not vary significantly before and after leaching, see Figure 7 and Figure 10.

**Figure 11:**
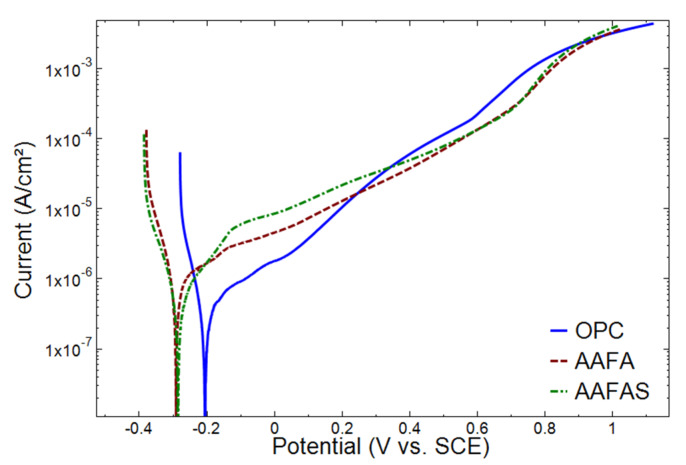
Potentiodynamic polarization curves of steel reinforcement embedded in OPC, AAFA, and AAFAS concretes after leaching.

PDP diagrams of the tested steels in OPC, AAFA, and AAFAS concrete are shown in Figure 11. The steel samples embedded in OPC concrete showed more noble potential than AAFA and AAFAS samples. Furthermore, this anodic shift in OPC concrete was accompanied by a lower corrosion current than in AAFA and AAFAS concrete. This response is common under passive conditions.

**Figure 12:**
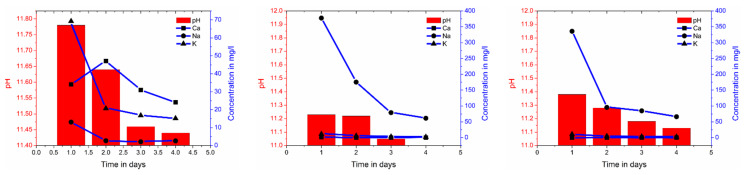
Changes in chemical composition and pH of water in which the leaching samples were stored: OPC (**left**), AAFA (**middle**), AAFAS (**right**).

To allow a more detailed analysis, both the pH values and chemical composition of the deionized water were monitored after different time intervals during the leaching test. The pH in the storage water decreased with time. The OPC had the highest pH and the AAFA the lowest pH at the beginning and the end of the measurements (Figure 11). The differing amounts of slag increased the pH by max. 0.2. The *Ca* content in the OPC water was very high because of the free *Ca(OH)_2_* in the pore solution. Due to the Portland cement clinker composition (see Table 1), the *K* content was much higher than the *Na* content. In AAFA and AAFAS, only high *Na* content from the activator was determined. The decrease of the pH value and alkali metals as well as alkaline earth metals is symptomatic of the leaching process (Figure 12).

#### 2.3.2. Comparative µXCT Investigations after Carbonation and Leaching Tests

**Figure 13:**
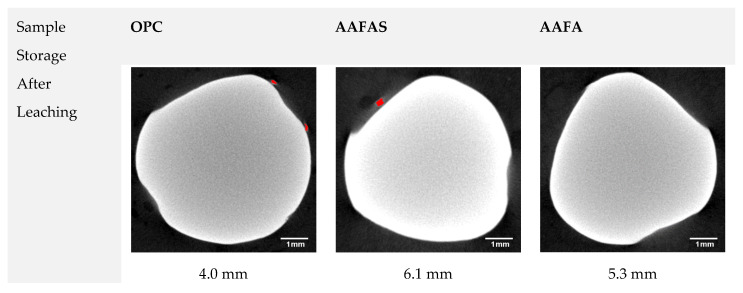

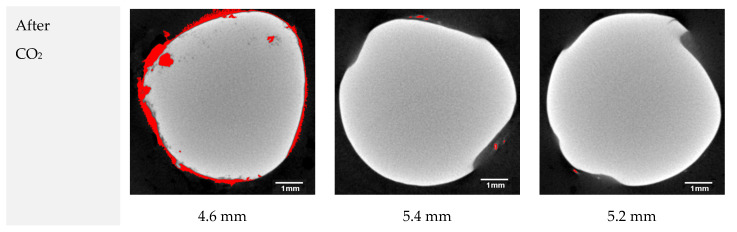
Micro X-ray computer tomography (µXCT) images of steel reinforcement cross sections in the mortar samples with the smallest mortar cover (see values). The red area shows new reaction products with a lower density than non-corroding steel in the transition zone between steel and mortar matrix after leaching and accelerated carbonation (*CO_2_* chamber).

An increase of voxels with a lower density (Figure 13) than steel and a higher density than mortar (maybe iron products) was significantly visualized only at end of the steel electrodes in the OPC sample, which was stressed by *CO_2_*. In both OPC samples, single disconnected micro cracks with a width of <0.03 mm, a length of <1 mm and a tangential orientation were visualized. The crack width was significantly lower in view of the crack width limitation for XC2 to XC4 (w_k_ = 0.2 mm according EN 206-1, w_k_ = 0.3 mm according EN 1992-1-1). In addition, the actual distance between the steel bars and the mortar surface is not a constant value because the ribbed steel bars do not have a constant cross section, the steel bars were not 100% parallel to the mortar surface, and the mortars have single air voids on the surface or steel. For these reasons, the actual distances were measured by µXCT (see Figure 13).

**Figure 14:**
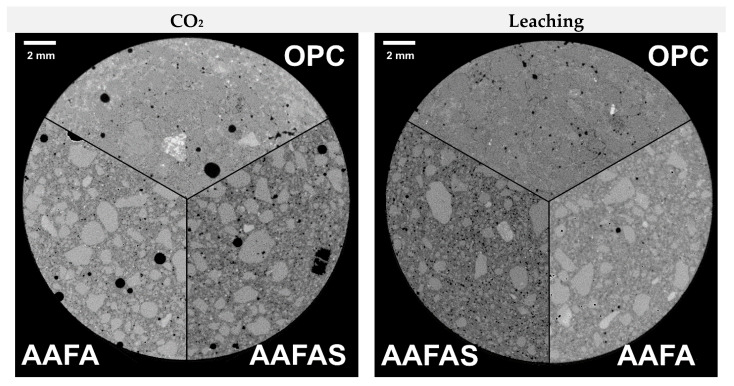

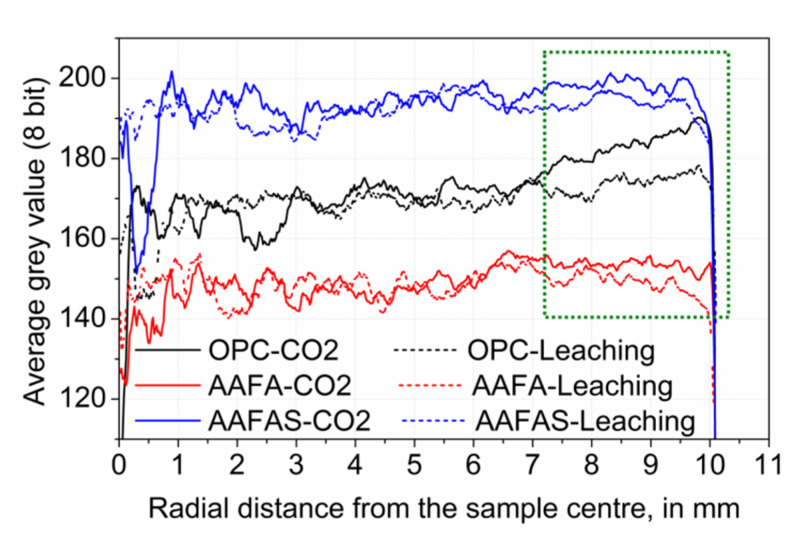
µXCT images of the cross sections after accelerated *CO_2_* exposure (**left top**) and leaching (**right top**); The graphs show radial plot profiles of >1000 cross sections referred (by inter standard) grey values (**down**).

Only the OPC mortar, which was stored in the *CO_2_* chamber, showed increased grey values at a depth of approximately 3 mm from the sample surface (Figure 14, left top). In contrast, both GP samples had a constant grey value distribution over the full cross section (Figure 14). The constant grey level gives evidence that the X-ray power was sufficiently high for radiography. In contrast to carbonation, only the leached AAFA samples showed a decrease in the grey values near the surface (green frame in Figure 14, depth 1 mm).

## 3. Discussion

### 3.1. Eluate Tests

The apparent paradox of decreasing pH value despite increasing sodium hydroxide content is well known in the fields of cement chemistry [59,60,61], concrete durability (e.g., Alkali-Silica-Reaction—ASR [62,63]), chemical sedimentology and glass investigation [64]. With a decreasing level of order in the single phases, the tendency to dissolve increases. For example, *SiO_2_* phases with a high level of order (e.g., rock crystal) have very low solubility, whereas the X-ray amorphous *SiO_2_*-rich parts in fly ash will dissolve very quickly with a proportional pH value. In the case of ASR, different kinds of silica (*SiO_2_·nH_2_O*) (This is the generic denomination of solved silicas. The water content *nH_2_O* does not be for crystal water.) with different water contents will be produced which absorb *OH^-^* on the surface (hydrogen bonds, see Equation (1)). The alkaline (*Na^+^*, *K^+^*) and earth alkali ions (*Mg^2+^*, *Ca^2+^*) balance the different *H_2_SiO_4_* species [63]. Similar to ASR, the elements *Si*, *Ca*, *Al*, and *Mg* are resolved by the alkali activation [65] first. Then the alkaline/earth alkali cations work as network changing components for the negative *AlO_4_* as well as *SiO_4_* tetrahedra during the geopolymerization, and the hydroxides are connected in form of hydrogen bonds in C-A-S-H/N-A-S-H phases [66,67,68]. Thus, the eluate results show at first a consumption of *OH^−^* by the solution as well as a silica production process. This reaction effect decreases the alkalinity in the first days. This behavior differs widely from normal Portland cement hydration and explains why the pH indicator test is not a good tool for the evaluation of carbonation in geopolymer systems.

SiO_2_ + 2OH^−^ → H_2_SiO_4_^2−^ H_2_SiO_4_^2−^ + 2Na^+^ → Na_2_SiO_3_ + H_2_O (1)

The eluate tests showed an increase of pH level with increasing *Ca* content in binder. Even a low *Ca* content (<5 w.-% respect to alumosilicate) interferes in the geopolymerization process, because of the high affinity of calcium towards silicate [69]. If the pH is higher than 12 and *Ca* ions are available, more C-A-S-H than N-A-S-H will be formed [70,71]. It is also known from zeolite synthesis (crystalline types of N-A-S-H, see also e.g., [72]) that sodium can be replaced by calcium and the number of O-H bonds can be changed by the pH and the free positive ions in the liquid. For example: if the *Ca* content increases in the liquid, more hydroxyl sodalite (HS) with lower O-H-bonds [73] than zeolite P will be formed [74]. It is possible that *Ca* can work more effectively as an ion exchanger than *Na* because of their double charge and the lower O-H-bonds can give rise to a higher pH value.

### 3.2. Concrete Carbonation Tests under Standard Climate Conditions

The carbonation rate of the OPC samples decreases with age (Figure 2) because the resulting calcium carbonates reduce the number of connected capillary pores and therefore gas permeability allowing *CO_2_*/*H_2_O* ingress [75]. In addition, the alkalinity in the OPC microstructures increases by means of cement hydration (*Ca(OH)_2_*, free sodium/potassium ions) only in the first days to a range between 13.5 to 13.9 [22].

According to current understanding [22,43,44,45], only the pore solution of GP and not the strengthening N-A-S-H can react with *CO_2_*. With the addition of slag, the calcium concentration increases and therefore C-A-S-H can be formed in addition to N-A-S-H. With increasing slag content, the compressive strength and at the same time the total porosity decreases along with the pore size. Nedeljković [45] has shown that the pH in the pore solution increases and the carbonation rate decreases with increasing slag content. This effect is confirmed by the measured carbonation rate under normal conditions (Figure 2) and the measured pH in the pore solutions (Figure 6).

The reflex at 29.4–29.5 2Ɵ can be allocated to calcite, which means that C-A-S-H was formed beforehand. The small shift of the reflex at 29.4 to 29.5 2Ɵ (see Figure 3, AAFAS diffraction patterns) can be a proof of the ion exchange of calcium by magnesium [76], which can be related to the *Mg* content in the amorphous portion of the slag (Table 1). On the basis of Vegard’s law, the ratio between *Mg* and *Ca* can be calculated as approximately 1.8/98.2. In comparison to calcite, the solubility of this carbonate phases would be lower [77,78]. The so-called “low magnesian calcite” (LMC, see Figure 3) in AAFAS can be the result of the carbonation of C-A-S-H, which is only described by [38,39,44,45] for AAS. Similar to OPC, the higher volume of calcite or LMC compared to educts (strengthening C-A-S-H), the low solubility of LUM compared to sodium carbonate minerals in GP, and furthermore the higher rest alkali produced by the unreacted activator (due to the higher reactivity of blast furnace slags compared to fly ash, see also higher natron content, see Figure 3) are possibly the reasons why the carbonation rate of AAFAS was lower than in AAFA. Nonetheless, the low number of phases, which can react with *CO_2_*, is one reason why the carbonation process of GP is much faster than in OPC. The theory must be proved for GP in a next step by means of pore size distribution measurements using Mercury Intrusion Porosimetry (MIP) and permeability measurements before and after carbonation and leaching.

Another main reason is the fact that the carbonation process of GP cannot be separated from the geopolymerization process, which also consumes *OH^−^* (see Section 4.1) [79]. This behavior is the reason that pH values decrease in carbonated (Figure 6) and non-carbonated (epoxy covered) samples with time (Figure 7). The pH values determined for GP are comparable with those in other studies based on alkali-activated metakaolin (10.5–12.0 after year) [22] or alkali-activated fly ash (12.0–11.7 after 90 °C [80]). The decreasing pH level with higher *CaO* content is explained by the ion exchange and the different kinds of alumosilicate (see Section 3.1). According to Temuujin et al. [81], porosity in alkali-activated fly ashes may be higher due to the addition of calcium, and therefore the effect on the alkalinity of changing phases would be also significant.

**Figure 15:**
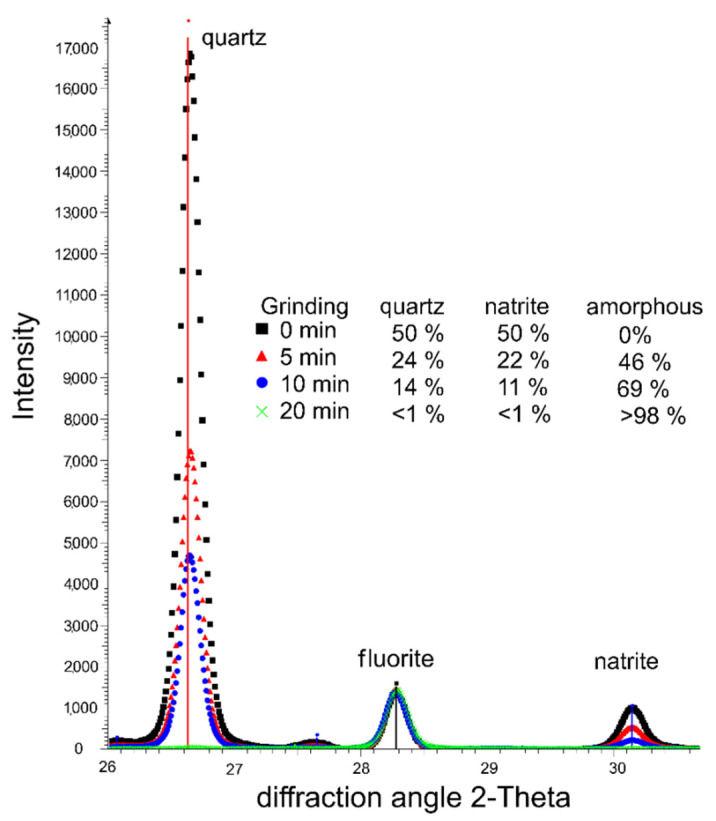
Effect of the network level of minerals (main reflexes of quartz, natrite) and the grinding process on XRD analysis in a simple mixture series (fluorite was the internal standard, which was added after grinding).

Radiographical identification of calcite in hardened cement paste [82] or in AAS [38,39,45] using XRD is much easier than it is for sodium carbonate minerals like natrite (Na_2_CO_3_) or natron (Na_2_[CO_3_]·10H_2_O). As the number of network levels (low-symmetric crystal structures) are much lower, the number of reflexes for the sodium carbonates are higher, and the intensity of each reflex in the diffraction patterns is much lower (Figure 3). This problem is aggravated by the fact that differences in the Mohs hardness between sodium carbonates (Mohs scale hardness 2) and other inert minerals like maghemite, mullite, or quartz (Mohs scale hardness 6–7) in the fly ash-based GP are greater than with calcite (Mohs scale hardness 3), C-S-H (Mohs scale hardness 3–4), and ettringite (Mohs scale hardness 2.5–3) in OPC systems. XRD measurement normally uses powders, which are normally generated by milling from solid samples, and the different hardness of the phases means that the sodium carbonate crystals sizes will faster decrease during the milling process than minerals with a higher hardness. The result is an increase in the X-ray amorphous portion. Nevertheless, the hardness effect was much lower than the number of network levels (see Figure 15).

Unlike calcite (*CaCO_3_*), natrite (*Na_2_CO_3_*) shows a hygroscopic behavior and its water solubility is very high (natrite 21.5 g/100 mL vs. calcite 0.67 mg/100 mL). Therefore, only natron (Na_2_[CO_3_]·10H_2_O) and no natrite could be determined in the hardened binder pastes, by XRD (Figure 3). The higher water solubility results in a high mobility in the microstructure and gives rise to a high sensitivity against leaching. The resulted sodium hydroxide is maybe the reason why a clear pink layer is visible near the surface when the pH indicator phenolphthalein was applied (Figure 1). The water content in the phenolphthalein solution (90%) dissolved the solid sodium carbonates (higher solubility compared to calcium carbonates) when the solution was sprayed. As a consequence, the pH rises due to dissociated sodium hydroxides (see Equation (2)). This behavior can be also an explanation for high pH values near the surface of the AAB concretes (10.5–11.5). Therefore, the phenolphthalein solution test approach is not useful for GP concretes/mortars as a method for rating the carbonation-induced reinforcement corrosion potential. The clearly differentiated behavior of the sodium carbonates in AAB concretes entails electrochemical investigation in order to verify the steel corrosion protection. The constant pH > 10 in the carbonated areas favor the application of steel in GP.

Na_2_CO_3_ + H_2_O → NaHCO_3_ + NaOH (2)

### 3.3. Accelerated Carbonation Tests on Mortars with Steel Reinforcement

The overall impedance values give indications for the corrosion resistance of the steel concrete in different concrete mixtures. It was clear that in case of AAFAS, the steel reinforcement showed less corrosion resistance. This fact was mainly related to the change in the expanded passive film properties in different concrete admixtures. On the basis of the EIS data and XRF analysis (Table 1), it can be surmised that the protective capacity of AAFAS pore solution arises from the aluminates and silicates, which form on the steel surface [83]. Farhana et al. [56] explained that a strong and adherent silicate membrane on the steel surface will be formed in GP concretes despite the comparatively low pH (11–12.5).

Similar to the unstressed samples, the highest impedance after the accelerating carbonation was measured in AAFA samples (Figure 8). The results are confirmed by Badar et al. [43], who determined a lower corrosion risk in low-*Ca* fly ash than in high-*Ca* fly ash. It can be concluded from the data in Figure 9 that at high frequency limit, the OPC samples present the highest impedance. These high values of impedance in OPC concrete were mainly related to the reduced alkalinity. This was not observed in GP concretes (AAFA and AAFAS), where the impedance values were approximately one order of magnitude lower than for OPC. This response is in agreement with the data obtained under controlled conditions (Figure 7), the potentiodynamic polarization curves (Figure 9), and the corrosion products detected by µXCT (Figure 13).

The quantitative changes of impedance document the influence of the carbonation process by means of barrier properties and alkalinity. The resulting pH values in the concrete cubes show a higher carbonation rate for GP than for OPC samples (see Figure 1). This effect could be the result of the lower content of phases in GP (compared to OPC-concrete, Figure 3), which can carbonate [43] the high mobility of the sodium carbonates (Figure 12), the geopolymerisation process (Figure 4 and Figure 5), and the lower buffer effect arising from the high solubility of the alkali compounds (in contrast to *Ca(OH)_2_* in OPC).

The radial plot profiles of the cross sections (Figure 1, green frame) show an increase in the grey values (equivalent to the density) only in the OPC sample, at a depth of approximately 3 mm after the accelerated carbonation. Therefore, the reduction of permeability by the larger volume of calcium carbonates in OPC is much greater than it is for sodium carbonate hydrates like soda (*NaHCO_3_•10H_2_O* = 196 cm^3^/mol) in GP: Theoretically, the influence decreases at higher *CO_2_* concentration or temperature because of the lower molecular mass of the different sodium carbonate hydrates like Nahcolith (*NaHCO_3_* = 38.66 cm^3^/mol), Trona (*Na_3_(HCO_3_)(CO_3_)•H_2_O* = 105 cm^3^/mol) or Thermonatrite (*Na_2_(CO_3_)•H_2_O* = 55 cm^3^/mol) [84]. It may be that the creation of low sodium hydroxide by dissolution of the sodium carbonates (Equation (2)) has an influence on the lower potential of steel corrosion in carbonated geopolymer. This assumption is confirmed by the pH development as shown in Figure 1 and Figure 5.

The EIS response of concrete samples exposed to leaching (Figure 10) showed similar responses to the control and carbonation samples (Figure 8). However, no corrosion initiation could be detected using electrochemical methods or µXCT (Figure 13) after leaching, and all samples showed higher impedance magnitude than in control and carbonation conditions.

This response reflects the passivation properties of steel reinforcement embedded in OPC, AAFA, and AAFAS concrete. As mentioned above, the steel reinforcement in the case of AAFA concrete presents the highest impedance magnitude and relatively highest capacitive EIS, where the phase angle reached values more than 80°.

These differences in the electrochemical response after leaching can be related to the variation in the chemical composition and the pH of the pore solution. The rapid decline of the sodium in the storage liquid from the AAFA and AAFAS samples (Figure 12) may be the result of the higher mobility of this element caused by the smaller ionic bond, the smaller atomic mass and the higher solubility of the solid phase. According to Nguyen and Škvára [85], the leaching rate of *Na^+^* ions increases with decreasing pH in the eluate. According to Fick’s law, leaching rate decreases with time because of the decreased difference between eluate and eluent. In contrast to GP, the main part of calcium in OPC results from *Ca(OH)_2_*, which is dissolved in the pore solution or exists as a solid phase (portlandite). This buffer effect leads to a slow decrease in alkalinity. When the leaching process is combined with carbonation, the buffer effect by sodium carbon hydrates will also decrease because of the high solubility of these carbonates.

## 4. Materials and Methods

### 4.1. Materials 

For the experimental study, a low calcium coal fly ash (according to EN 450-1) and a mix of low calcium coal fly ash and blast furnace slag (blast furnace slag according to EN 15167-1) were activated by sodium hydroxide together with a sodium silicate solution. For comparative purposes, specimens cast with ordinary Portland cement (OPC), CEM I 42.5 R according to EN 197-1, were also examined. The chemical composition, the X-ray amorphous portion, the loss on ignition (LOI), and the specific surface area (SSA) of the raw materials are given in Table 1. The phase composition of the three raw materials is published in [12].

Quarzitic river aggregates with a maximum grain size of 16 mm were used for the concrete and the same sand with a maximum size of 2 mm was used for the mortars. The workability of the fresh concretes and mortars was adjusted by the addition of superplasticizers based on ligninsulfonate (LSN) to have a similar spread flow class (F3 high –F4 low according EN 12350-5).

### 4.2. Mixture Proportions

The tested concrete and the mortar mix designs are presented in Table 2. The OPC concrete mix design as a reference system meets all requirements for all exposure classes in EN 206-1 and has a very high alkalinity. The mix designs of both geopolymer-concretes achieved an adequate strength level for most normal concrete constructions. The mortar scale was necessary for micro X-ray computer tomography (µXCT) and electrochemical investigations in order to ensure easy handling and a suitable sample size. The mortar mixed design is similar to the concrete with the exception of the coarse gravel aggregates.

The solid concrete components (with the exception of slag) were dry mixed in a 60-litre compulsory concrete mixer for 30 s. After the addition of activators and water, the mixing was continued for another 60 s before the slag was added. The sodium lignosulfonate (LSN) superplastizer is one of a few admixtures that work under this very high alkalinity at the beginning of the geopolymer process [12,58]. The significant amount for alkali-activated fly ashes with ten percentage mass (wt%) of blast furnace slag (AAFAS) was necessary to increase the workability and particularly the setting time, because the glass content in the blast furnace will dissolve very quickly. The total mixing duration amounted to approx. 240 s. The mortars were mixed in accordance with EN 196-1. All samples (concrete as well as mortars) were heat-treated at 35 °C for 24 h after demolding and afterwards stored under standard climate conditions (20 °C/65% rH/0.04% CO_2_). In order to assess the performance and possible construction applications, the compressive strength was determined in accordance with EN 12390-3. The properties of the fresh and hardened concretes are presented in Table 3.

### 4.3. Test Procedure

#### 4.3.1. Carbonation Resistance of Concretes under Normal Condition

At first, concretes cube specimens (100 × 100 × 100 mm^3^) based on an ordinary Portland cement (OPC) and the two alkali-activated binders (alkali-activated fly ashes (AAFA), AAFAS) were prepared and stored under standard climate conditions (20 °C/65% rH/0.04 % CO_2_) to test the carbonation resistance. After 1, 3, 7, 28, 56, 112, 365, and 730 days the cubes were split in two parts. The changes in the alkalinity of the first series were estimated by spraying a phenolphthalein solution (10%) on the surface of one part of the split cube, taking into account DIN CEN/TS 12390-10. The pH of the second cube part was analyzed at different depths (0–5 mm/5–50 mm). The 0–5 mm depth should be a relevant carbonation depth after few years for a normal OPC concrete. The second depth from 5–50 mm was chosen to identify the influence of hydration as well as geopolymerization process.

The collected concrete samples were grinded and then milled to a particle size lower than 0.125 mm. In each case, 10 g of the resulting powder was dissolved in 50 mL demineralized water and the pH value was determined after 24 h of mixing into account TGL 33422/13.

To allow a more detailed analysis, the crystalline reaction products of the carbonated concrete specimens were analyzed by X-ray diffraction (XRD). To visualize the X-ray phase-specific effects, XRD analysis of 50% quartz (SiO_2_) and 50% sodium carbonate (*Na_2_CO_3_*) was performed after varying grinding duration.

**Figure 16:**
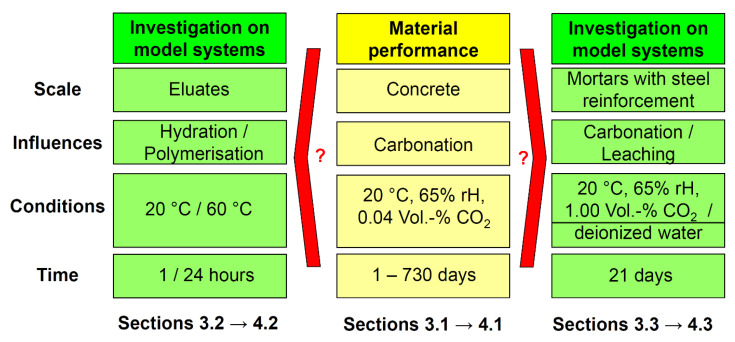
Schematic visualization of the test approaches.

Because of the special behavior of the carbonation products (Section 4.2), the hardening cement pastes from the concretes were produced exclusively with hand mixers. After 28 days of ageing the samples, they were grinded manually as well as completely into powders with a particle size <0.125 mm. After storage for 112 days under standard climate conditions, the powders were analyzed using XRD (Figure 16).

#### 4.3.2. Eluate Monitoring Tests

Firstly, eluate monitoring tests were carried out with the raw binder material fly ash (FA) as well as with a mix of blast furnace slag and fly ash (FAS) to determine the development of alkalinity until the reaction mechanism of the binders started. For the eluate tests, different solutions were prepared with a precisely defined amount of materials (4 g) dissolved in demineralized water (100 g), followed by slow titration of sodium hydroxide solution. The pH value and the temperature were measured after 1 h and after 24 h. In order to accelerate the process, the eluates were heated to 60 °C in the second test series.

#### 4.3.3. Accelerated Carbonation and Leaching Tests on Mortars with Steel Reinforcement

In order to investigate the potential for steel corrosion, electrochemical tests were carried out on reinforced mortar samples with the same binders. The plain steel electrodes (low carbon S235JR, ribbed surface) were ultrasonically degreased in ethanol, rinsed with distilled water, and then dried before being embedded in the three different mortars (see Table 2). The steel bar surface area exposed to the concrete was defined using a shrinkage tube, where the active surface area of the tested steels was 15 cm^2^.

**Figure 17:**
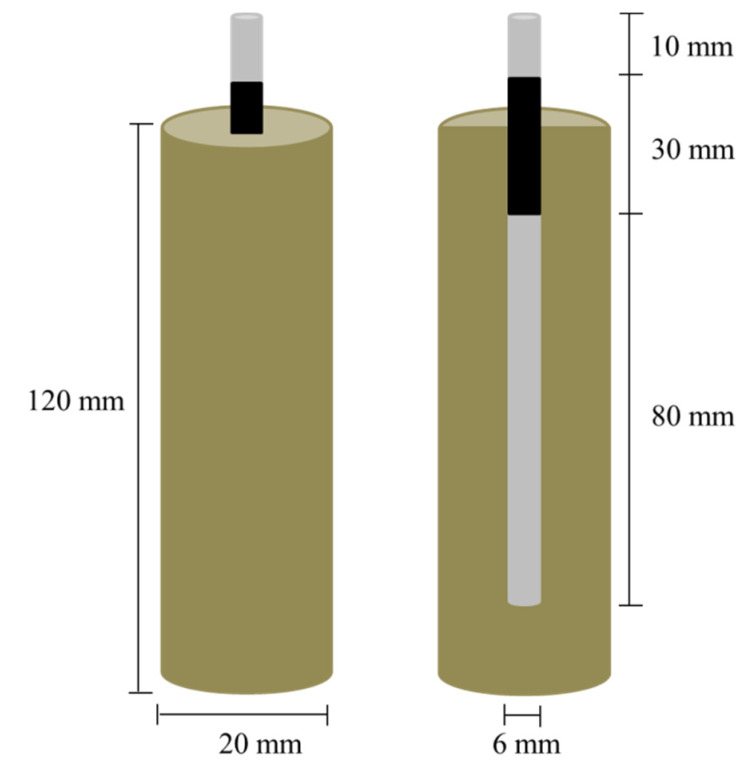
Schematic illustration of the test set up for electrochemical investigations.

The electrochemical response of the reinforced concrete samples was evaluated in cylindrical specimens. The schematic illustration of the test set up is shown in Figure 17.

As with the concrete, the mortar samples were heat-treated at 35 °C for 24 h and were protected against atmosphere as well as leaching by the mold under standard climate conditions (20 °C/65% rH) until they were 28 days old. After removal, the sample batch was subdivided into two series.

Regarding carbonation conditioning, the first series of mortar samples was exposed under lab conditions for 7 days, followed by an accelerated carbonation process. In this case, carbonation was performed by additional exposure to 1% CO_2_ and 66% rH for 21 days. The total curing time was 56 days (28 days of ageing + 7 days lab conditions + 21 days accelerated carbonation).

The mortar samples in the second series were totally immersed in deionized water for 4 days in order to allow the analysis of the leaching process in parallel to carbonation.

Three electrochemical cells were used to electrochemically monitor the embedded reinforcements in mortar. A saturated calomel electrode (SCE) was used as the reference electrode, and MMO Ti- mesh as the counter electrode. Furthermore, two identical cylindrical mortar specimens (replicates) were immersed in tap water and used as reference electrodes for each exposure scenario.

### 4.4. Methods

#### 4.4.1. Micro-X-Ray Computer Tomography (µXCT)

A micro-X-ray computer industrial tomograph (FhG, Dresden, Germany) was used to visualize the effects of steel corrosion and the change of density in the microstructure after leaching and accelerated carbonation. The µXCT was also used to determine the mortar covering.

The μXCT was performed with an X-ray power of 17.8 watts (140 kV/140 μA), a copper filter (0.5 mm), and a step size of 0.2/360 (1800 positions). The copper filter was necessary to minimize the characteristic radiation and to reduce the beam hardening effects on both the sample surface and on the steel reinforcement. The voxel edge length was maintained at 31.4 μm (V = 30,959 μm^3^).

The data set was analyzed using VGStudio (version. 2.2, Volume Graphics) and ImageJ (version 1.47, National Institute of Health) after a grey value calibration based on an internal standard [86]. The grey values are the result of the X-ray absorption. As density and atomic mass increase, X-ray absorption also increases and therefore the grey values become brighter. The corrosion products on the steel electrodes were visualized with ImageJ based on lower grey values within the cross sections. For better representation, the corrosion products are colored red. The transformation in the mortar microstructure caused by accelerated *CO_2_* exposure as well as by leaching was analyzed in single cross sections using an ImageJ plugin called “radial profile plot” (author Paul Baggethun, Pittsburgh, PA, USA).

#### 4.4.2. X-Ray Diffraction (XRD)

A D8-Discover (Bruker AXS, Karlsruhe, Germany) X-ray diffractometer equipped with a VANTEC-500 area detector was used for XRD analysis. The instrument operated with Cu-Kα radiation (λ = 1.5418 Å) at 40 kV and 40 mA. It was utilized to characterize the crystalline products formed during carbonation on hardened binder paste produced in accordance with concrete mix designs. The dried powders with a particle size of <0.125 mm were mixed with fluorite as internal standard and measured in a 2Ɵ range of 5–70°. The quantification of the mineral phases as well as the calculation of the amorphous portion was carried out by using Topas (v.4.2; Bruker AXS, Karlsruhe, Germany) Rietveld refinement software.

#### 4.4.3. Electrochemical Response of Reinforcement

The progressive changes in the electrochemical response of the steel electrodes exposed to OPC, AAFA, and AAFAS concrete were tested after two different curing times; (i) before exposure to accelerated carbonation or leaching processes. The mortar samples had an age of 35 days (28 days curing and 7 days under lab conditions); (ii) at the end of the carbonation and leaching test (i.e., sample age was 56 days). The accelerated carbonation of mortar samples was performed for 21 days in a climate chamber at 1% CO_2_ and 66% RH. In parallel, reinforced mortar samples were totally immersed for 4 days (sample age 52 days) in deionized water in order to evaluate the effect of leaching on the electrochemical response of the reinforcement. For the leaching test samples, 0.5 L deionized water was used for two replicate samples, with the deionized water changed every day. The pH value and the elemental analysis for the collected solution were measured every day.

The electrochemical behavior of carbon steel embedded in different mortars (OPC, AAFA, and AAFAS) was monitored by measuring the Open Circuit Potential (OCP), Electrochemical Impedance Spectroscopy (EIS), Polarization Resistance (Rp), and Potentiodynamic Polarization curves (PDP).

Electrochemical impedance spectroscopy (EIS) was carried out using Metrohm Autolab-Potentiostat PGSTAT30. EIS measurements were recorded under potentiostatic control at the corrosion potential, from 50 kHz down to 10 mHz. An AC perturbation voltage signal of 10 mV (rms) amplitude was applied. At the end of the test, the specimens were subjected to PDP in a potential range from −0.2 to +1.2 V vs. E_corr_, with a scan rate of 1 mV/s.

## 5. Conclusions

From the current study, the following conclusions can be drawn:

Eluate tests exhibit a very fast (minutes to hours) consumption of OH in the first hours by the reaction mechanism. The pH values in carbonated and non-carbonated GP concretes approach 11–12 in the first 112 days. With increasing *Ca* content in the raw material, the pH increases in the non-carbonated and in the carbonate microstructure.

The phenolphthalein solution test approach is not useful for rating the carbonation-induced reinforcement corrosion potential in GP concretes/mortars after more than one year, because the decrease in alkalinity is a combination of reaction mechanism, carbonation, and leaching effects. The applied phenolphthalein solution dissolved the sodium carbonates. Sodium hydroxides occur and the increasing pH led to just colored single areas but not to layers. Therefore, a clear zoning cannot be detected after one year within GP concretes/mortars using the phenolphthalein solution test.

Carbonation tests on hardened cement paste indicated that sodium carbonate hydrates like soda and natrite are the result of the carbonation of only *NaOH* from the pore solution, but not from the strengthening N-A-S-H. The identification of these carbonation products in concrete or mortar by XRD is difficult because of the low content, in particular the network level, the hardness of the minerals, and the overlaying with inert phases (e.g., maghemite, natrite) from the fly ash.

With increasing *Ca* content within the raw material (for example by dosing of blast furnace slag), more C-A-S-H will be formed in the non-carbonated area, and in the presence of *CO_2_*, C-A-S-H will carbonate to calcite.

Tomography measurements showed increasing grey values only in carbonated OPC, but not near the surface after carbonation in the GP samples. These values indicate higher density in the voxels and therefore possibly a reduction of small pores (at the resolution of V = 30,959 μm^3^) by carbonation.

In the next step, detailed investigations of the changing permeation and pore size distribution caused by leaching and CO_2_ are necessary for these geopolymer systems. Furthermore, more fundamental investigation of the electrochemical action of steel in AAB must be carried out. A better rapid test for the evaluation of the corrosion process of AAB systems needs to be developed.

## Figures and Tables

**Table 1:** Chemical composition based on X-ray fluorescence spectroscopy (XRF) analyses in wt%, loss on ignition (LOI) in wt%, specific surface area (SSA) in m^2^/g and X-ray amorphous portion on the basis of the X-ray diffraction (XRD) Rietveld analyses in wt% Abbreviations: FA, fly ash; OPC, ordinary Portland cement; S, blast furnace slag.

Raw Material	SiO_2_	Al_2_O_3_	Fe_2_O_3_	CaO	MgO	SO_3_	K_2_O	Na_2_O	LOI	SSA	X-Ray Amorphous
**FA**	52.85	25.57	9.41	3.26	1.74	0.48	2.42	1.44	1.55	1.06	63.2
**S**	35.35	11.18	0.57	40.33	6.83	2.69	0.68	0.18	-	3.00	98.8
**OPC**	20.10	4.82	3.20	63.5	3.46	1.27	0.99	0.21	2.22	3.11	4.0

**Table 2:** Concrete mix design in kg/m^3^ and mortar mix design in g. Abbreviations: AAFA, alkali-activated fly ashes; AAFAS, alkali-activated fly ashes with ten percentage mass (wt% of blast furnace slag; LSN, sodium lignosulfonate.

Scale	Components	Concrete in kg/m^3^			Mortar in g		
Components		OPC	AAFA	AAFAS	OPC	AAFA	AAFAS
**Binder**	CEM I 42.5 R	360	-	-	569	-	-
	Fly Ash	-	488	443	-	724	654
	Blast furnace slag	-	-	45	-	-	73
**Activator**	Sodium silicate (Na_2_O/SiO_2_ = 0.9)	-	43	43	-	182	182
	NaOH (30 %)	-	20	20	-	99	99
**Water**	Total water	162	142	142	256	210	210
	Additional water	162	15	15	256	23	23
	w/b-value	0.45	0.27	0.35	0.45	0.27	0.35
**SP**	LSN	0.7	2.5	12.2	-	1.5	5.0
**Aggregates**	Sand 0–2 mm	735	689	691	1203	1032	1040
	Gravel 2–8 mm	551	517	518	-	-	-
	Gravel 8–16 mm	551	517	518	-	-	-

**Table 3:** Properties of the concretes.

Properties	Units	OPC	AAFA	AAFAS
**Fresh concretes**				
Spread flow (EN 12350-5)	mm	460	580	480
Air content (EN 12350-7)	vol.-%	2.6	2.2	2.6
Bulk density (EN 12350-6)	kg/m^3^	2.358	2.325	2.341
**Hardened concretes (cubes 100 × 100 × 100 mm^3^)**				
Compressive strength 28d	N/mm^2^	49.4	19.4	30.5
Compressive strength 56d	N/mm^2^	56.5	27.0	34.7
Compressive strength 112d	N/mm^2^	61.9	30.4	36.3
